# Editorial: Big scientific data analytics on HPC and cloud

**DOI:** 10.3389/fdata.2024.1353988

**Published:** 2024-02-20

**Authors:** Jianwu Wang, Junqi Yin, Mai H. Nguyen, Jingbo Wang, Weijia Xu

**Affiliations:** ^1^University of Maryland, Baltimore County, Baltimore, MD, United States; ^2^Oak Ridge National Laboratory (DOE), Oak Ridge, TN, United States; ^3^University of California, San Diego, La Jolla, CA, United States; ^4^Australian National University, Canberra, ACT, Australia; ^5^The University of Texas at Austin, Austin, TX, United States

**Keywords:** scientific discovery, high-performance computing (HPC), big data analytics (BDA), cloud computing, science and engineering

In an era characterized by an exponential surge in data generation across scientific and engineering domains, the convergence of big data analytics and high-performance computing (HPC) alongside cloud computing stands as a pivotal enabler of monumental discoveries. This Research Topic is a testament to the transformative power held within this convergence, shedding light on the boundless possibilities and challenges in leveraging these distributed environments for data-driven scientific exploration.

The manifold submissions to this Research Topic encapsulate a collective effort aimed at unraveling the complexities of big scientific data analytics. They encapsulate not just the strides made in harnessing HPC and cloud computing for data analysis but also the novel methodologies, algorithms, and frameworks that propel scientific discovery at an unprecedented scale.

## Fostering cross-disciplinary insights

One of the standout features of this Research Topic is the cross-disciplinary nature of the insights offered. From atmospheric science to healthcare and beyond, the diverse spectrum of contributions underscores the universality of big data analytics in advancing knowledge boundaries. Scalable algorithms tailored for scientific data, automated analytics workflows, and the deployment of analytics tools on HPC and cloud environments illuminate a path toward multidimensional exploration.

## Performance optimization and innovation

Integral to this discourse are the discussions on performance optimization. The intricate interplay between hardware configurations, network setups, and algorithmic innovations has been a focal point. Whether it is exploring GPU acceleration, distributed AI applications, or the burgeoning landscape of edge device analytics, each article unravels layers of optimization crucial for achieving efficiency and scalability in data analysis.

## Toward sustainable scientific practices

Beyond showcasing cutting-edge technological advancements, this issue also champions the ethos of reproducibility and benchmarking. The emphasis on reproducible analytics on HPC and cloud, alongside benchmarking for data science at scale, sets a precedent for fostering robust and transparent scientific practices essential for progress and credibility within the field.

## The path ahead

As we delve into the myriad articles encapsulated within this issue, it is imperative to acknowledge that this is not a culmination but a continuum of exploration. The wealth of knowledge encapsulated within these submissions paves the way for future endeavors, sparking conversations around the evolution of data analytics, the integration of emerging technologies, and the ever-expanding horizons of scientific inquiry.

In the pursuit of advancing scientific discovery through big data analytics on HPC and cloud environments, this assemblage of scholarly contributions serves as a cornerstone—a testament to the relentless pursuit of knowledge and the unwavering spirit of innovation.

Among many manuscripts submitted, four papers were accepted by the Research Topic. We will briefly explain their work:

1. “*Opportunities in open science With AI,”* authored by Wang is a PERSPECTIVE article discussing how big data and AI techniques are helping open science. With increasingly affordable computation, openly available big datasets, and advances of artificial intelligence (AI) technologies, more and more scientists are embracing open science by sharing their data and code. Such trends also have a positive impact on the researchers themselves by increasing citations of their work.
2. “*Examining the relationship between big data analytics capabilities and organizational ambidexterity in the Malaysian banking sector*,” authored by Aziz and Long, demonstrated that data analytics capabilities could positively influence two contradictory aspects of organizational ambidexterity. The study is based on the survey results from interviewing 162 bank managers in Malaysia. The dynamic capability view is adopted as the grounded theory of the research to study the linkage of big data analytics capabilities and ambidexterity in the banking sector.
3. “*CRMnet: A deep learning model for predicting gene expression from large regulatory sequence datasets,”* authored by Ding et al., introduced a novel deep-learning model – CRMnet – to predict gene expression in saccharomyces cerevisiae. Leveraging recent large datasets measuring gene expression, CRMnet outperforms existing benchmarks. The model's interpretation techniques, including saliency maps, successfully identify informative genomic regions. The study also compares practical training times on a large compute cluster, emphasizing CRMnet's efficiency for similar datasets.
4. “*Real-time arrhythmia detection using convolutional neural networks*,” authored by Vu et al., proposes a CNN-based approach for detecting arrhythmia from ECG images that can be performed in real time. This approach is shown to deliver accurate and efficient detection. The work presented here shows the potential for enabling in-home, real-time heart monitoring, which can be an important tool in long-term cardiac care.

## Author contributions

JiaW: Writing—review & editing. JY: Writing—original draft. MN: Writing—review & editing. JinW: Writing—review & editing. WX: Writing—review & editing.

